# Mapping sources of noise in an intensive care unit

**DOI:** 10.1111/anae.14690

**Published:** 2019-05-07

**Authors:** J. L. Darbyshire, M. Müller‐Trapet, J. Cheer, F. M. Fazi, J. D. Young

**Affiliations:** ^1^ Nuffield Department of Clinical Neurosciences University of Oxford UK; ^2^ National Research Council of Canada Ottawa ON Canada; ^3^ Institute of Sound and Vibration Research Southampton University Southampton UK

**Keywords:** alarms, ICU environment, monitors, noise

## Abstract

Excessive noise in hospitals adversely affects patients’ sleep and recovery, causes stress and fatigue in staff and hampers communication. The World Health Organization suggests sound levels should be limited to 35 decibels. This is probably unachievable in intensive care units, but some reduction from current levels should be possible. A preliminary step would be to identify principal sources of noise. As part of a larger project investigating techniques to reduce environmental noise, we installed a microphone array system in one with four beds in an adult general intensive care unit. This continuously measured locations and sound pressure levels of noise sources. This report summarises results recorded over one year. Data were collected between 7 April 2017 and 16 April 2018 inclusive. Data for a whole day were available for 248 days. The sound location system revealed that the majority of loud sounds originated from extremely limited areas, very close to patients’ ears. This proximity maximises the adverse effects of high environmental noise levels for patients. Some of this was likely to be appropriate communication between the patient, their caring staff and visitors. However, a significant proportion of loud sounds may originate from equipment alarms which are sited at the bedside. A redesign of the intensive care unit environment to move alarm sounds away from the bed‐side might significantly reduce the environmental noise burden to patients.

## Introduction

Excessive ambient noise in hospitals adversely affects patients’ sleep and recovery, causes stress and fatigue in staff and hampers communication. In critical care areas, disruption of patients’ sleep patterns may contribute to the development of delirium 1, 2. Patients who experience delirium in hospital may have longer hospital stay and ongoing cognitive impairment after discharge home 3.

The US Environmental Protection Agency (USEPA) suggests sound pressure levels in hospitals should be limited to 45 decibel (dB)‐equivalent continuous sound level (LAeq) during the day, and 35 dB at night 4. The World Health Organization (WHO) advises sound pressure levels in hospitals should not exceed 35 dB 5. For neonatal intensive care units (ICUs), an upper limit of 45 dB is recommended 6, with limits on transient loud sounds 7, 8.

In a previous study of environmental noise in five general adult ICUs in the Thames Valley region of the UK, average sound pressure levels always exceeded 45 dB, and for 50% of the time exceeded 52–59 dB in individual units 9. Although the WHO and US EPA guidelines are probably unachievable in any acute care area of a hospital, some reduction from these high levels should be possible. A preliminary step would be to identify the location and sources of noise.

As part of a project investigating initiatives to reduce noise in a single ICU, we installed a microphone array system in a single bay containing four beds in an adult general ICU. This continuously recorded the location, sound pressure levels and weighted ‘loudness’ values of environmental sound. This study summarises the results over one year.

Noise is usually described as ‘unwanted sound’. In this article, we refer to ‘sound’ as raw, objective values (reported as sound pressure levels, measured in decibels); ‘noise’ signifies a subjective response. Features of sound, such as volume, frequency (Hertz), duration and time of occurrence are likely to affect subjective impression of sound. ‘Loudness’ is a weighted value (also measured in decibels) that allows the subjective perception of sound to be described and quantified.

## Methods

This study focussed on environmental monitoring and did not involve patient recruitment or the use of any identifiable information. The system recorded sound pressure levels only; no audio recordings were made. The local ethics policy does not require formal review/approval for studies based on environmental data as they contain no identifiable information that can be traced back to individuals.

We used a four‐bed bay in the general ICU at the John Radcliffe Hospital in Oxford (UK) for the study. In addition to beds and associated equipment, the bay contained equipment racks, trolleys and a built‐in counter (nursing station) that housed two telephones and a computer. Two individual patient rooms opened into the bay on one side.

The bay was of standard construction, with painted plasterboard walls, fitted vinyl flooring and a ceiling of suspended perforated fibro‐cement tiles. One wall had large windows and a fire exit.

In October 2016, we installed a sound monitoring system into the ceiling of this bay. This consisted of four arrays of 16 microphones that used beam forming technology to measure horizontal locations and sound pressure levels of the five loudest sounds above 35 dB for each time‐point. The system had a temporal resolution of between 2 s and 4 s, depending on the computational processing burden. The sound pressure levels and location of the five loudest sounds above 35 dB were logged for each time‐point. The absolute values of sound pressure levels were then weighted to calculate ‘loudness’ (the loudness value being the subjective perception of sound). These weighted values were calculated using the Zwicker method, which forms the basis of the ISO standard method for calculating loudness (ISO 532‐1: 2017) 10. The details of the system have been reported elsewhere 11. The area monitored was 14.1 × 12.7 m.

Data for this study were collected from 7 April 2017 to 16 April 2018 inclusive. Data for a whole day were available for 248 days out of the total 381 days. Data were analysed using MATLAB R2018a (MathWorks, MA, USA).

The decibel level and location data for the entire period were summarised by counting the number of times one of the five loudest sounds originated from each location in the bay, and by averaging the loudness. The resolution of the location in both X and Y axes was approximately one cm. The count and average loudness data were indicated using colour, and plotted on a heat map overlaying a floor plan of the bay.

Temporal changes in loud sounds were summarised by similar plots of counts for one h corresponding to the loudest (19h00–19h59) and quietest (04h00–04h59) one‐h periods identified in the earlier study 9.

The sound level monitoring system was installed as part of a range of measures to make the ICU quieter. One element of this was the development of a wall‐mounted display with the capacity to show the location and volume of sound sources to ICU staff in real time. Staff were involved in the design of this, and were shown the screen interface for short periods of time to allow them to evaluate and comment upon the utility of the data displayed. Their feedback guided design and feature iteration. The display did not run in real time during the project outside of these evaluation sessions. Frames captured from one of the display prototypes were combined to produce a time‐lapse video to show the variability in sound sources and loudness over an hour.

## Results

The system recorded 248 complete days of data out of a total 381 days between 7 April 2017 and 16 April 2018 inclusive. Missing days were when the system was inactive for technical development or repairs. In all, there were 12,245,708 data triplets (X position, Y position, sound pressure level) included in the analysis dataset.

During the study period, occupancies for beds A3–A6 were 85.8%, 85.7%, 87.6% and 74.6%, respectively, with an average of 83.4%. The median (IQR [range]) length of stay was 3.1 (1.7–6.6 [0.0–66.8]) days. The number of staff members in the bay was determined by the number of patients and their care requirements, and so varied during the study. The handover periods for the nursing staff were constant (07h30–08h00 and 19h30–20h00) during the study. Ward round times and durations varied with the overall number of patients in the ICU.

**Figure 1:**
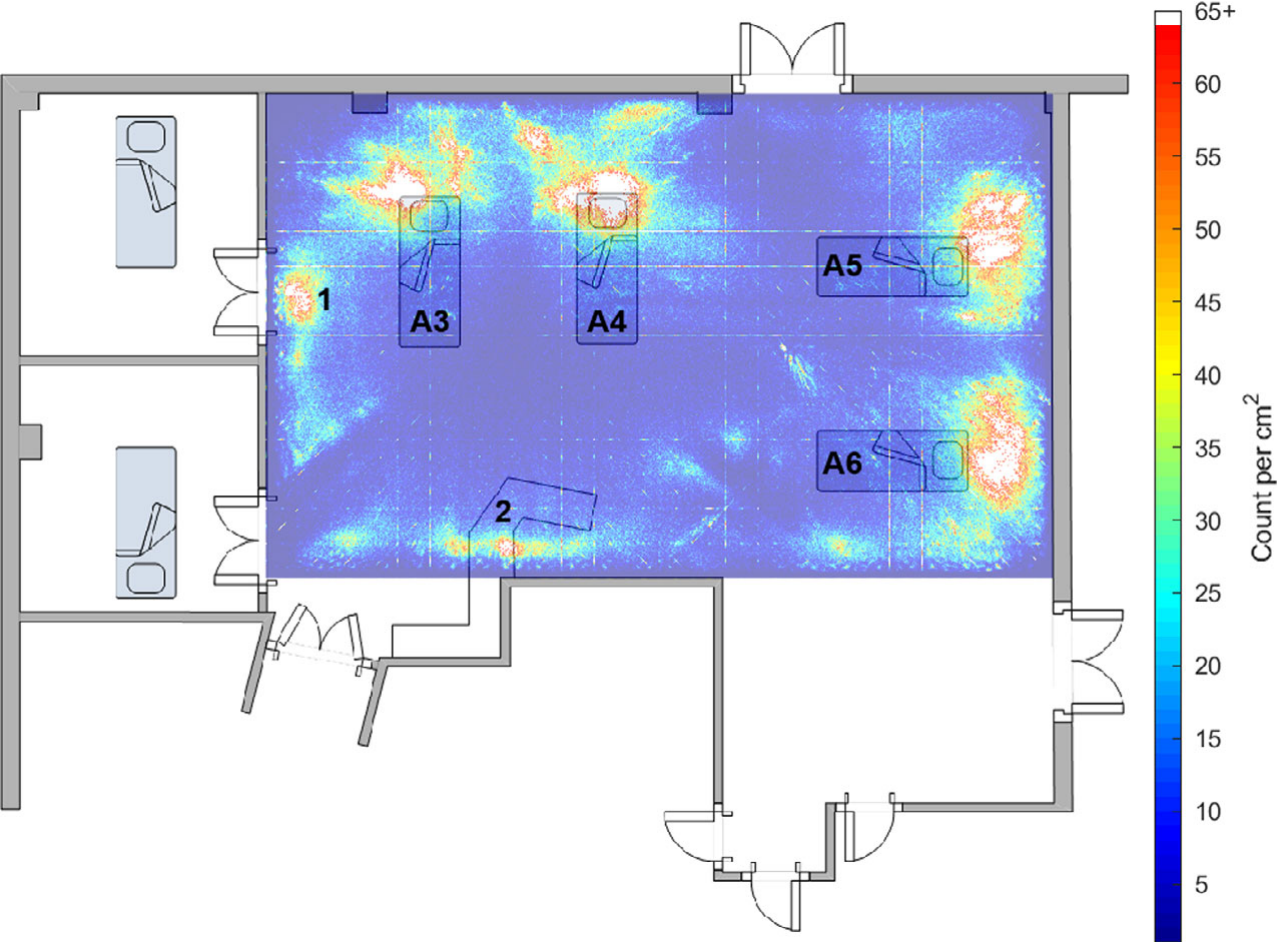
A heat map of the frequency with which one of the five loudest noises above 35 dB originated from each 1 cm^2^ of the intensive care unit bay for the 249‐day study period, superimposed on a floor plan of the bay. The position and identifier for each of the four beds in the bay is shown, as well as the work bench position. The area of noise marked ‘1’ is outside the side room that was preferentially used, and shows where conversations between staff about the patient in the side room commonly took place. The areas of noise marked ‘2’ correspond to the positions of the telephones. This can be interpreted as a map of the ‘noisiness’ of areas of the bay. The grid lines are an artefact of the computational methods.

Figure 1 shows the frequency with which one of the five loudest noises in any 2–3‐s epoch occurred in each 1 cm^2^ of the monitored area. ‘Hotter’ colours (reds and yellows) indicate areas where loud noises were more frequent. The heat map was superimposed on a floor plan of the ICU bay. The uppermost wall in the graphic contained the windows and fire exit and faced southeast. This graphic can be interpreted as an average ‘noisiness’ map for the bay.

The beds were labelled A3–A6. In all bed spaces, the physiological monitors were mounted on the patients’ right, level with or slightly behind their heads. The ventilators were positioned in front of the monitors. Beds A3, A4 and A5 had a clear area of high sound level corresponding to the monitor/ventilator position. This was less clear for A6, the least occupied bed during the study period. The area of loud noise indicated by ‘1’ was outside the door of the side room that was preferentially used, as it had the best natural light. Area ‘2’ was at the nursing station where the telephones were situated. The horizontal and vertical lines are artefacts of the computational methods.

**Figure 2:**
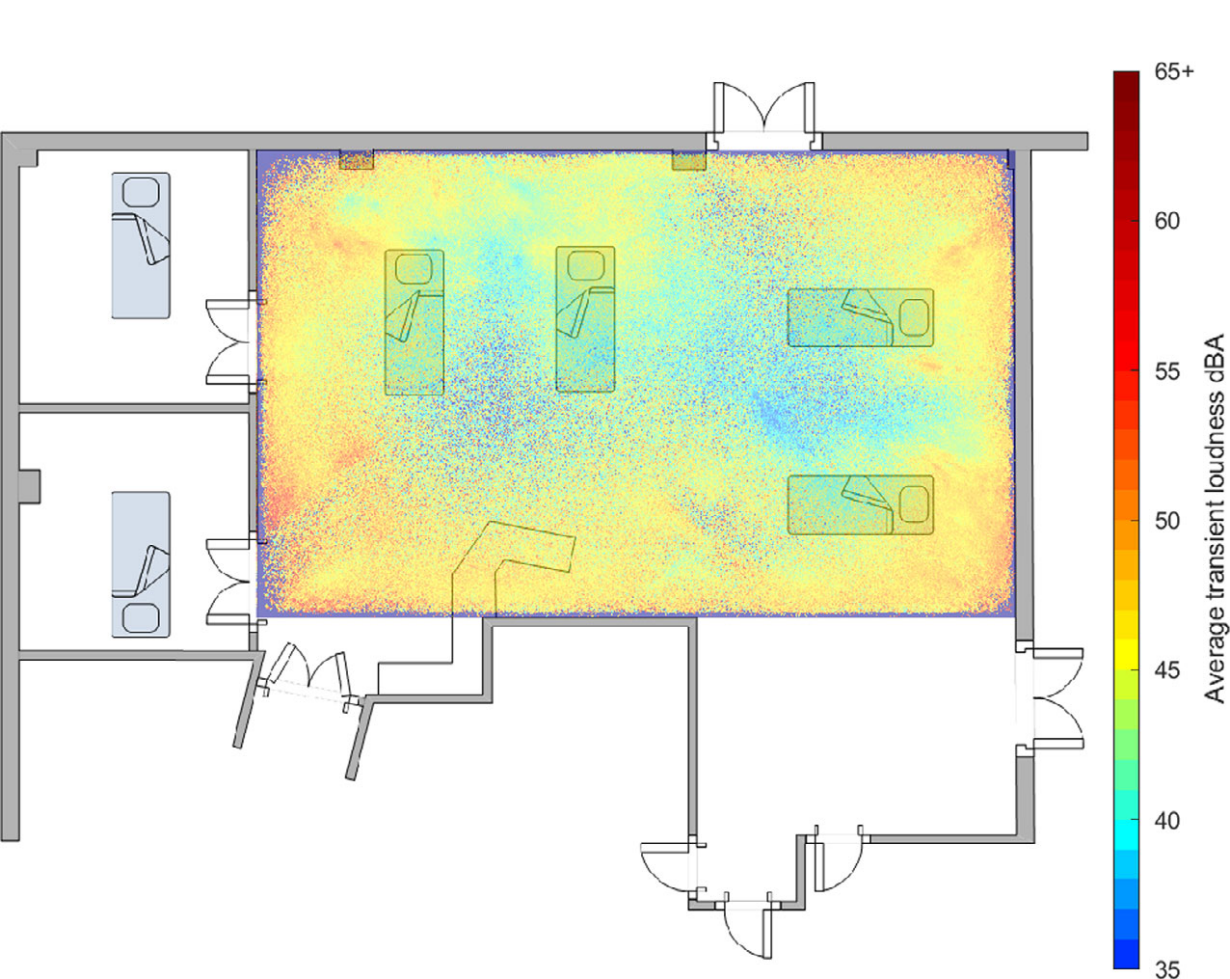
A heat map of the average loudness of noises above 35 dB originated from each 1 cm^2^ of the intensive care unit bay for the 249‐day study period, superimposed on a floor plan of the bay. The position and identifier for each of the four beds and the work bench in the bay is shown.

Figure 2 shows the averaged loudness value of the five loudest sounds above 35 dB in any 2–4‐s epoch that occurred in each 1 cm^2^ of the monitored area. ‘Hotter’ colours (reds and yellows) indicate the areas with higher averaged loudness. The plot does not contain any information on the number of loud sounds in any one area. This graphic can be interpreted as showing how loud, on average, noises were that originated from each 1 cm^2^ of the monitored area. Louder noises originated from room peripheries and were generally centred on the head area of bed spaces. Noise originating from the centre of the room was on average less loud.

**Figure 3:**
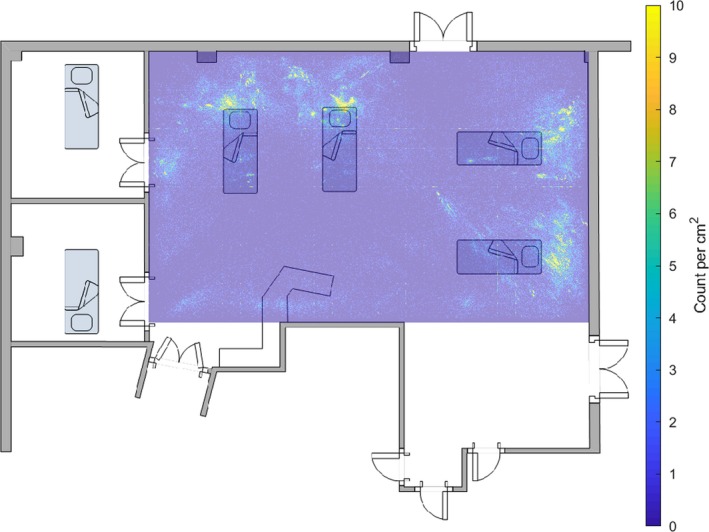
A heat map of the frequency with which one of the five loudest noises above 35 dB originated from each 1 cm^2^ of the intensive care unit bay between 19h00  and 20h00 for the 249‐day study period, superimposed on a floor plan of the bay. The position of each of the four beds in the bay is shown, as well as the work bench position. The grid lines are an artefact of the computational methods. The counts are considerably lower than in Fig. 1, as this plot only represents 1/24th of the data in Fig. 1.

Figure 3 shows a heat map of the frequency with which one of the five loudest sounds above 35 dB originated from each 1 cm^2^ of the ICU bay between 19h00 and 20h00. Counts were considerably lower than in Fig. 1, as this plot only represents about 1/24th of the data in Fig. 1.

**Figure 4:**
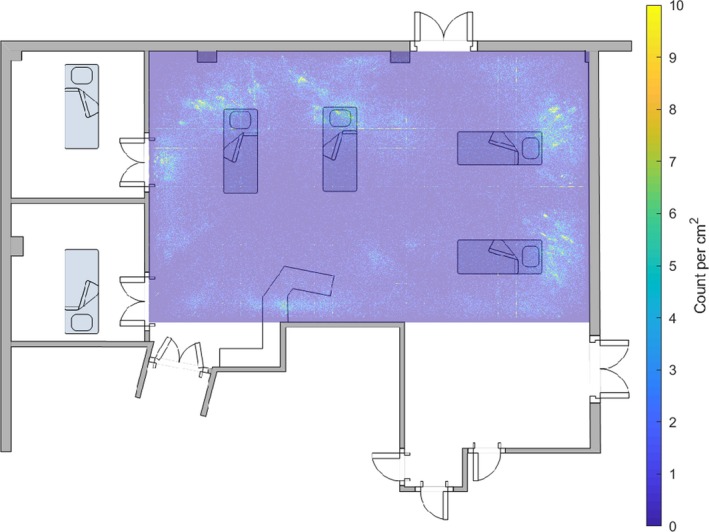
A heat map of the frequency with which one of the five loudest noises above 35 dB originated from each 1 cm^2^ of the intensive care unit bay between 04h00 and 05h00 for the 249‐day study period, superimposed on a floor plan of the bay. The position of each of the four beds in the bay is shown, as well as the work bench position.

Figure 4 shows a similar heat map for 04h00–05h00.

The additional file (see also Supporting Information, Video S1) shows the locations of the five loudest sources of noise above 35 dB repeatedly superimposed on the floorplan of the ICU to form a time‐lapse movie. The movie represents the period from 09h30 to 10h30 on a weekday in spring 2017. The loudness was encoded as the diameter of a red circle centred on the location of the noise source. Data from the two previous epochs are indicated by successively paler circles. The pale grey circle on the floor plan indicates where the bay co‐ordinating meeting commonly occurred, and where clinical teams tended to congregate when reviewing patients in the side rooms and bed A3. From about 09h30–09h40 high noise levels from closely spaced locations (likely speech) were visible in this area. Transient noises adjacent to beds were most likely to be equipment‐generated, for example, alarms from physiological monitors, ventilators and infusion pumps. Some frames (e.g. in the last second of the movie) had fewer than five noise sources above 35 dB.

The system recorded the raw sound pressure levels, loudness values, frequency spectrum and location of sounds, and could not be used to determine the originating source of the sounds directly. A machine learning approach was used to try to separate the sounds into classes (e.g. ‘alarms’, ‘speech’ or ‘other’), but did not achieve reliable identification. However, an earlier phase of our project included an ethnographic study conducted between November 2014 and July 2015 12. This indicated that noise from equipment was pervasive. The volume of equipment‐related noise was consistently high, and activity around patient bed spaces often led to alarms triggering, which were not silenced until patient care activity was concluded.

## Discussion

Hospital noise is increasing. A systematic review suggested that the A‐weighted SPL (corrected for the human hearing range) is increasing by 0.26 dB annually, and levels are such that both speech comprehension and communication are compromised 13.

The novel and striking result from this study was that most high levels of noise originated from extremely limited areas. Some of these areas were where staff conversations commonly occured, such as the nursing station or the area where daily group discussions take place. However, it is clear that the majority of loud sounds originated very close to patients. Some of this would have been appropriate communications between the patient, their caring staff and visitors. However, there was a clear indication that much loud sound originated from physiological monitors and ventilators, sited near patients’ ears. These devices generated very little sound, except when alarms were activated. Alarms were loud (> 50 dB) and within a frequency range that humans find disturbing. Alarms of all kinds by design have sound characteristics that activate human sensitivities. The frequency range of the patient monitor alarms in the ICU (2.5–3.15 kHz) is similar to both a human scream and a baby's cry. Although this may be ideal for attracting the attention of staff members, it is far from ideal for patient rest and comfort.

The alarms on the physiological monitors default to standard volume settings on power‐up, and could be adjusted as required by the nursing staff. In practice this rarely occurred. Similarly, although it was possible to adjust the volume settings for ventilator and infusion pump alarms, these were not adjusted routinely. By default, the ventilator alarms increased in volume with increased urgency.

As reported elsewhere 12, 14, our project utilised the AEBCD method 15 to design and deliver a number of interventions to reduce noise levels in the ICU. Briefly, these included soft‐close plastic‐lidded bins, better day/night differentiation, and alarm management guidelines that recommended ‘personalising’ alarm parameters and adjusting volume according to the wider environmental sound level. We also created a training package that delivered an online module and an experiential simulation session, giving staff members a ‘patient experience’ of the ICU. Interim results of this phase of our project indicated reduced SPL in the ICU by approximately 4 dB after four months (measured April 2016), suggesting that some knowledge transfer occurred as a result of the relatively simple intervention programme. Despite this, and the apparent sustained reduction in overall SPL, noise levels in close proximity to the patients remained high.

In the early hours of the morning, when patients should have been sleeping, the staff tried to keep disturbances to a minimum. However, in spite of the fact that the bay seemed quieter, and previous environmental monitoring reported average sound pressure levels reached a nadir at this time, Figs. 3 and 4 suggest the number of transient high‐level noises were similar in the early evening and early morning. If average sound levels decreased but the number of loud transient noises remained comparable, the reduction in disturbance to patients may have been less than the change in average SPL suggests.

Intensive care units are particularly noisy areas of hospitals 1. Neonatal units have average sound pressure levels of 48–61 dB for up to 95% of the time 16, 17, 18, 19, paediatric units average 53–73 dB 20, 21, 22, 23, 24 and adult units are 53–59 dB 25, 26, 27, 28, 29. The unit at the John Radcliffe Hospital when measured in 2012 had daytime averages of 58 dB at the desk and 60 dB adjacent to the patient 9. There seems to be considerable concordance between average sound pressure levels recorded in ICUs in different healthcare systems, and across levels measured in the wider hospital 13.

Excessive noise in ICUs disrupts patients’ sleep 1, 27, 30, increases sleep medication use 2, sedation use 24 and the incidence of delirium 2. Ambient sound markedly reduces the intelligibility of speech in acute care areas 13, which can contribute to avoidable errors in care. To be intelligible, speech needs to be about 15 dB above ambient sound pressure levels. Conversations, therefore, increase sound pressure levels which aggravates the problem (Lombard effect 31). High ambient sound levels also have a range of deleterious non‐auditory health effects on staff 32.

Acute care areas in hospitals rely heavily on alarms and pagers to signal urgent situations. It is, therefore, unsurprising that observational studies on ICUs identify these as major disruptive noise sources. Nearly 80% of disruptive noises are generated by monitor or ventilator alarms and speech 33, 34. However, a significant proportion of the noise coming from speech is not required for patient care 1, 2, and almost 90% of alarms from physiological monitors are ‘false positives’, with no patient benefit 35.

The standard measure of environmental noise, the average daily sound pressure levels value (LAeq24), is insufficiently detailed to enable targeted noise‐reduction interventions in an ICU. This may be one of the reasons why the majority of sound reduction studies conducted in ICUs have not resulted in significant change 36, 37, 38. This study was designed to identify spatial positioning of sound sources within an ICU. This would enable interventions to be focussed on areas of high noise levels, which might lead to meaningful reduction in overall (averaged) sound levels.

Sound pressure levels increase logarithmically with proximity to the source of the noise. There is no reason for alarms from physiological monitors, ventilators or any other piece of equipment to be generated next to a patient. The primary function of alarms is to alert staff to a possible clinical problem. Arguably, there is no reason why patients need to hear these alarms at all. Hearing alarms may cause patients distress or contribute to disorientation 39. With modern digital electronics and wireless networks, an ICU free from intrusive alarms is possible, although there are considerable commercial, regulatory and safety hurdles to overcome before this can be achieved 40. In addition, changing a technology that has been in use for decades requires careful planning and detailed assessment of working patterns that may have evolved alongside the technology 41. During the early phase of this project, the use of body‐worn haptic alerts linked to monitoring equipment was suggested as an alternative to acoustic alarms. The nurses felt this approach would remove the multiple layers of redundancy that an alarm heard by all staff added to their practice, and would not consider this technological adaptation. Although they acknowledged the distress that alarms might cause, patients also expressed concern that urgent clinical situations might go unattended if this form of technology were adopted.

At the very least, conversations not directly involving patients or their visitors should occur away from the bed‐side, and ideally outside the unit, if possible. A body‐worn electronic communicator has been successfully trialled in an ICU in the USA 13, although the major gain was a reduction in overhead paging calls, which are not used routinely in the UK.

The sound location system revealed that the majority of noise in this four‐bed ICU bay was generated immediately adjacent to patients’ ears. This proximity maximised the adverse effects of high environmental noise, levels for patients. Paying attention to the locations of significant high noise, as well as the overall sound level, might improve both the patient experience of their ICU admission, and the general working environment for staff.

The sound location system deployed for this study was both complex and expensive, and required minor building works to install. Although it would be ideal to repeat this work in other ICUs to test generalisability, this might not be practical. However, a simplified version of the system with single microphones near patients’ heads at each bed space would be quite feasible. This would enable unit‐wide evaluation of noise levels as experienced by individual patients.

## Supporting information


**Video S1.** Video of noise source location.
