# Supplementary material for: Mapping sources of noise in an intensive care unit
Source: Anaesthesia. 2019 May 7;74(8):1018–25. doi: 10.1111/anae.14690 (PMC6767712; doi:10.1111/anae.14690)
Supplement: Supplementary file 2 [file ANAE-74-1018-s002.docx]

**Additional material description**

**File name:**

am_noise.mpg

**File format:**

MPEG

**Title of data:**

Video of noise source location

**Description of data:**

The video file “am_noise.mpg” shows the location of the five loudest noise sources above 35dB superimposed at 6-9 second epochs on to the floorplan of the intensive care unit to form a time lapse movie. The loudness at each location is indicated by the diameter of a red circle centred on the noise. Data from two previous epochs are indicated by successively paler red circles. The movie covers the period from 09h30am to 10h30am on a weekday in the spring of 2017. In addition to the showing the location of the beds and desk there is a pale grey circle on the floor plan indicating where both the bay co-ordinating meeting occured (nursing staff) and where medical teams tended to congregate when reviewing patients in the side rooms and bed A3.
